# Intrafamilial phenotypic variation in spinocerebellar ataxia type 23

**DOI:** 10.1186/s40673-020-00117-x

**Published:** 2020-06-23

**Authors:** Shunichi Satoh, Yasufumi Kondo, Shinji Ohara, Tomomi Yamaguchi, Katsuya Nakamura, Kunihiro Yoshida

**Affiliations:** 1 Department of Neurology, Nagano Red Cross Hospital, 5-22-1 Wakasato, Nagano, 380-8582 Japan; 2 Department of Medicine (Neurology & Rheumatology), Shinshu University School of Medicine, 3-1-1 Asahi, Matsumoto, 390-8621 Japan; 3 Department of Neurology, National Hospital Organization, Matsumoto Medical Center, 2-20-30 Muraicho Minami, Matsumoto, 399-8701 Japan; 4 Department of Neurology, Iida Hospital, 1-15 Odori, Iida, 395-8505 Japan; 5 Department of Molecular Genetics, Shinshu University School of Medicine, 3-1-1 Asahi, Matsumoto, 390-8621 Japan; 6 Center for Medical Genetics, Shinshu University Hospital, 3-1-1 Asahi, Matsumoto, 390-8621 Japan; 7 Department of Brain Disease Research, Shinshu University School of Medicine, 3-1-1 Asahi, Matsumoto, 390-8621 Japan

**Keywords:** Spinocerebellar ataxia type 23 (SCA23), Prodynorphin, Multiple system atrophy (MSA)

## Abstract

**Background:**

Spinocerebellar ataxia type 23 (SCA23) is an autosomal dominant cerebellar ataxia caused by pathogenic variants in the prodynorphin gene (*PDYN*). The frequency of *PDYN* variants is reportedly very low (~ 0.1%) in several ataxia cohorts screened to date.

**Case presentations:**

We found five cases of SCA23 in two families (mean age at onset: 37.8 ± 5.5 years; mean age at examination: 64.2 ± 12.3 years) with a novel *PDYN* variant (c.644G > A:p.R215H). We identified marked heterogeneity in the clinical features in Family 1: the proband showed clinical and neuroimaging features suggestive of multiple system atrophy with predominant parkinsonism (MSA-P). Conversely, the proband’s mother with the *PDYN* p.R215H variant had no subjective symptoms; she had not come to medical attention before our survey, although she showed apparent cerebellar atrophy on brain magnetic resonance imaging (MRI). The other two patients in Family 1 and a patient in Family 2 showed slowly progressive cerebellar ataxia.

**Conclusions:**

We here report two Japanese families with SCA23, one of which showed considerable phenotypic variation in affected members. Our findings support that SCA23 can phenotypically overlap with MSA.

## Introduction

Spinocerebellar ataxia type 23 (SCA23) is a rare form of autosomal dominant cerebellar ataxias (ADCAs) caused by mutations in the prodynorphin gene (*PDYN*) [1–5]. This rare disorder was originally identified in a Dutch family: the affected family members presented with late-onset (> 40 years), slowly progressive, pure cerebellar syndromes with hyper-reflexia [6]. Since its discovery, eight SCA23 disease-causing variants in *PDYN* have been identified, six of which lie exclusively within the PDYN Dyn A and Dyn B encoding regions (amino acid residues 207–236) that form the peptide known as “Bigdynorphin” [1, 2].

To the best of our knowledge, only 20 patients worldwide, including two Japanese patients in the same family, have been confirmed to carry disease-causing *PDYN* variants to date [1–6]. Here, we report another two Japanese families (family 1 and family 2) with SCA23 carrying a novel *PDYN* variant, p.R215H. Family 1 included three patients heterozygous and one patient homozygous for the *PDYN* variant: the proband exhibited clinical and neuroimaging features resembling multiple system atrophy with predominant parkinsonism (MSA-P).

## Case report

The clinical details of the patients from Families 1 and 2 are summarized in Table 1.

**Table 1:** Clinical and neuroimaging features of the patients with SCA23

Family No.	1	1	1	1	2
Pedigree number/Sex	II-1 (male)	II-4 (male)	II-8 (male)	I-2 (female)	II-1 (male)
Age at examination (years)	62	59	52	88	60
Age at onset (years)	45	36	40	-^a^	30
Disease duration (years)	17	23	12	-^a^	30
Initial symptom	Unsteadiness	Dysarthria	Dysarthria	-^a^	Dysarthria
Walking ability	Unaided	Wheelchair	Unaided	Crutches/cane	Unaided
SARA	5.0/40.0	19.0/40.0	9.0/40.0	14.5/40.0	7.5/40.0
Dysarthria	(++)	(+++)	(+)	(−)	(+)
EOM disorder	Saccadic	(−)	(−)	(−)	Gaze nystagmus
Tremor	Resting tremor(−)	(−)	Cerebellar tremor	(−)	Cerebellar tremor
Parkinsonism	(+)^b^	(−)	(−)	(−)	(−)
Deep tendon reflex	Increased (U/L)	Increased (L)	Increased (L)	Normal	Decreased (U/L)
Babinski sign	(−)	(−)	(−)	(−)	(−)
Muscle atrophy & weakness	(−)	(−)	(−)	(−)	(−)
Sensory deficits	(−)	(−)	(−)	NA	(−)
Urinary symptoms	(−)	(−)	(−)	(−)	(−)
<Brain MRI findings>					
Atrophy of the cerebrum	(−)	(−)	(−)	(−)	(−)
cerebellum^c^	(+)	(++)	(++)	(++)	(++)
brainstem (pons)	(−)	(−)	(−)	(−)	(−)
middle cerebellar peduncle	(−)	(−)	(−)	(−)	(++)
Hyperintense lateral putaminal rim	(+)	(−)	(−)	(−)	(−)
Hot cross bun sign	(−)	(−)	(−)	(−)	(−)
*PDYN* (c.644G > A) genotype	Heterozygote	Homozygote	Heterozygote	Heterozygote	Heterozygote

NA: Information not available. SARA: Scale for the Assessment and Rating of Ataxia. ^a^ This patient had no subjective symptoms and had never visited a neurology clinic before we performed a medical survey on the family members of the proband (II-1). ^b^ Parkinsonism means bradykinesia, small steps, and frozen gait. ^c^ All patients showed atrophy of both cerebellar hemispheres and vermis. (−): none, (+): mild, (++): moderate, (+++): severe, (U): upper extremities, (L): lower extremities

**Fig. 1:**
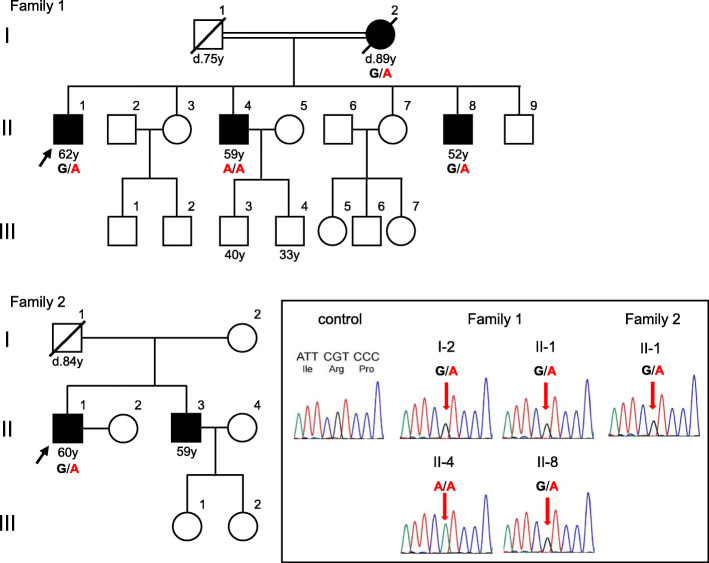
Pedigrees of Families 1 and 2 and Sanger sequencing for *PDYN*. The numbers below the symbols indicate the age (in years, y) at examination and the age at death (d) for the deceased individuals (indicated by the strikethrough). The probands are indicated by the arrows

**Fig. 2:**
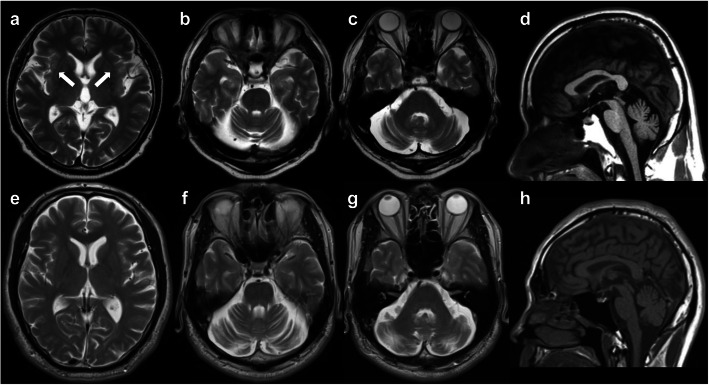
Brain MRI. (**a–d)**: Patient II-1 in Family 1 had mild atrophy of the cerebellar hemisphere and vermis. A hyperintense lateral putamen rim (arrows) was seen. (**e–h)**: Patient II-1 in Family 2 had moderate atrophy of the cerebellar hemisphere and vermis. No brainstem atrophy, hot cross bun sign, or atrophy/hyperintensities in the middle cerebellar peduncles was observed in either patient

**Fig. 3:**
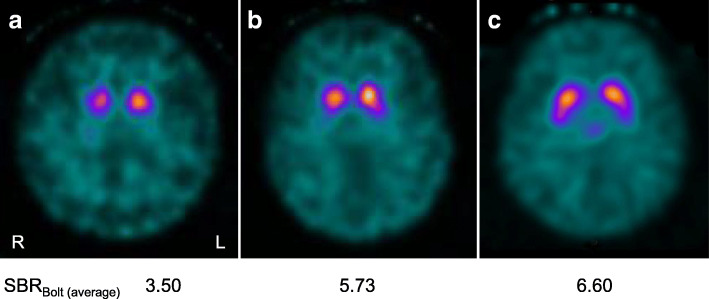
Dopamine transporter single-photon emission computed tomography using ^123^I-ioflupane. The proband (II-1) in Family 1(**a**) and an unrelated 60-year-old patient with Parkinson’s disease (**b**) both showed decreased bilateral striatal uptake of ^123^I-ioflupane, more marked in the putamen, while another unrelated 65-year-old patient with drug-induced parkinsonism (**c**) showed a normal pattern of uptake. The age-matched values for specific binding ratio (SBR) refer to 7.50 ± 1.35 (mean ± SD)

Family 1: The proband (Fig. 1, II-1) first noted an unsteady gait at 45 years-of-age. At ~ 58 years of age, he developed speech disturbance and right hand tremor. These symptoms gradually worsened, prompting him to visit a hospital at 62 years-of-age. A neurological examination revealed saccadic eye movements, scanning speech, hyperreflexia of all limbs, and parkinsonism such as bradykinesia, rigidity, frozen gait, and a resting tremor affecting both hands (R > L). He also complained of constipation, but did not have orthostatic hypotension or urinary incontinence. He could walk unaided and his ataxia was very mild [Scale for the Assessment and Rating of Ataxia (SARA) score: 5.0/40.0]. Brain magnetic resonance imaging (MRI) revealed mild atrophy of the cerebellar hemisphere and vermis, as well as a thin hyperintense lateral putaminal rim (Fig. 2 a-d). A hot cross bun sign and/or hyperintensities in the middle cerebellar peduncles were not evident. His parkinsonism responded slightly to levodopa (Levodopa/Carbidopa hydrate 300 mg/day) and other anti-parkinsonian drugs (Pramipexole Hydrochloride 1.5 mg/day and Selegiline hydrochloride 0.5 mg/day). Dopamine transporter single-photon emission computed tomography (DAT-SPECT) at 65 years-of-age showed severe bilateral nigrostriatal dopaminergic deficits bilaterally predominantly in the putamen (Fig. 3).

The younger brother of the proband (Fig. 1, II-4) noticed difficulty in speaking smoothly at around 36 years-of-age. A neurological examination at 37 years-of-age revealed scanning speech, dysmetria of the upper limbs, and increased deep tendon reflexes of the legs. A brain computed tomography scan showed cerebellar atrophy and he was subsequently diagnosed with spinocerebellar degeneration (SCD). At 59 years-of-age, he needed a wheelchair because of ataxic gait, and his dysarthria became very severe; his SARA score was 19.0/40.0. Brain MRI scan revealed moderate atrophy of the cerebellar hemisphere and vermis, but brainstem atrophy or abnormal signals, including a hot cross bun sign or hyperintensities in the middle cerebellar peduncles were not evident.

The proband’s youngest brother (Fig. 1, II-8) developed difficulty in speech and unsteadiness at 40 years-of-age. Brain MRI at 42 years-of-age revealed moderate cerebellar atrophy. A later neurological examination at 52 years-of-age revealed a cerebellar tremor and hyper-reflexia of the lower limbs. His gait was ataxic, but he could walk unaided; his SARA score was 9.0/40.0.

Their parents of these affected siblings were first cousins. Their mother (Fig. 1, I-2) was hospitalized for crowned dens syndrome at 88 years-of-age, and this was the first time she had undergone any neurological examinations. She also had chronic heart failure and osteoarthritis of the knee joints, but had no subjective complaints of cerebellar ataxia, and was almost independent in her daily life. However, her gait was ataxic and she needed a cane for walking; her SARA score was 14.5/40.0. Brain MRI showed moderate cerebellar atrophy. We assumed that she had cerebellar ataxia like her affected children, but the age of onset was ambiguous. She died of heart failure at 89 years-of-age. The father of the proband (Fig. 1, I-1) died of lung cancer at 70 years-of-age. Although his clinical details were not available, his close family members reported that he did not have dysarthria or a gait disturbance during his lifetime.

Family 2: The proband (Fig. 1, II-1) became aware of slurred speech at 30 years-of-age. At around 45 years-of-age, he noticed a disturbance of skillful movements of both hands that prompted a visit to hospital. Brain MRI scan revealed cerebellar atrophy and he was subsequently diagnosed with SCD that was treated with the thyrotropin-releasing hormone analog, taltirelin. A detailed neurological examination at 60 years-of-age revealed mild dysarthria, horizontal nystagmus, a cerebellar tremor affecting both hands, decreased deep tendon reflex without muscle atrophy or sensory disturbance. He could walk unaided and his SARA score was 7.5/40.0. Brain MRI identified moderate atrophy of the cerebellar hemisphere and vermis, but no atrophy or abnormal signals in the brainstem (Fig. 2e-h).

The proband’s brother (Fig. 1, II-3) exhibited speech disturbance from ~ 40 years-of-age, but his clinical details were not available. His father (Fig. 1, I-1) died from pneumonia at 84 years-of-age, while his mother (Fig. 1, I-2) had dementia and had been institutionalized in a nursing home. Neither parent was reported to have ataxic symptoms during their lifetime.

### Diagnostic evaluation

We performed a molecular diagnosis using genomic DNA extracted from a blood sample from each patient. Screening for common repeat expansions for ADCAs (SCA1, SCA2, MJD/SCA3, SCA6, SCA7, SCA8, SCA12, SCA17, and DRPLA) and SCA31 was negative for the proband in each family (II-1 in Family 1, II-1 in Family 2). We then performed targeted re-sequencing for both probands in each family using a TruSight One Sequencing Panel (Illumina) and a MiSeq benchtop sequencer (Illumina). Here we sequenced previously reported causative genes of autosomal dominant and recessive cerebellar ataxias.

We identified a c.644G > A:p.R215H variant in *PDYN* exon 3 in the proband of each family. Sanger sequencing confirmed that four patients (Family 1: I-2, II-1, and II-8; Family 2: II-1) were heterozygous for this variant, and one patient (Family 1: II-4) was homozygous for this variant (Fig. 1). This variant was registered in the Single Nucleotide Polymorphism database (dbSNP138) as rs201655505, with a minor allele frequency of 0.00003 according to the Exome Aggregation Consortium (ExAC) database [7]. Further analyses using SIFT (http://sift.jcvi.org/), PolyPhen-2 (http://genetics.bwh.harvard.edu/pph2/), and MutationTaster (http://www.mutationtaster.org/) predicted that this missense variant was deleterious. Furthermore, a different missense change at the same amino acid residue, p.R215C, has been demonstrated to be pathogenic [1]. Based on these data, we consider p.R215H as “likely pathogenic” according to the American College of Medical Genetics (ACMG) standards and guidelines [8].

## Discussion and conclusions

The clinical phenotype of SCA23 is highly variable, but its cardinal feature is a late onset, slowly progressive cerebellar ataxia [1, 6]. Interestingly, we found marked intrafamilial variation in the clinical and neuroimaging features in the affected members of Family 1. In particular, the proband (II-1) had suspected MSA-P before the diagnosis of SCA23 was made. Because this patient had no urinary dysfunction (incontinence, urgency, or incomplete bladder emptying) or orthostatic hypotension, he did not fulfill the second consensus criteria of probable or possible MSA [9]. Saigoh et al. reported the first Japanese case who had a hot cross bun sign and hyperintense lateral putaminal rim on brain MRI and suggested that the pontocerebellar tract or striatonigral tract may be damaged in SCA23 [5]. Together with the previous report [5], our cases suggest that the existence of a phenotypic overlap between SCA23 and MSA, both clinically and neuroradiologically.

On the other hand, the parents of the proband in Family 1 did not exhibit signs of cerebellar ataxia until they had reached an advanced age. This finding is in stark contrast to the fact that their affected children developed neurological disorders in the young-adult ages. The low penetrance rate for *PDYN* variants might have contributed to the marked intrafamilial variation observed in this family. Another possibility is that genetic anticipation occurred in this family, as seen non-repeat expansion mutations in *TMEM240* that underlie SCA21 [10, 11].

Family 1 consisted of patients who were heterozygous and homozygous for the variant. The age of onset for the homozygous patient (II-4) was a little younger than that for the heterozygous patients, and the ataxic symptoms were the most severe. As for the pathogenesis of SCA23, both ‘toxic’ gain of function [1, 12, 13] and loss of function [2] mechanisms have been postulated. Although the clinical difference between heterozygous and homozygous patients in Family 1 does not support either a ‘toxic’ gain-of-function or loss-of-function mechanism, it might suggest that the dosage effect of the variant or normal PDYN proteins is directly associated with the clinical severity of SCA23.

In conclusion, we identified two Japanese families with SCA23 likely driven by an underlying, novel *PDYN* p.R215H variant. The cardinal clinical feature of the patients was a slowly progressive cerebellar ataxia with young-adult onset. Importantly, one patient showed clinical and neuroradiological features similar to MSA-P.

## Data Availability

The data in this study are not publicly available as they could compromise the anonymity of the subjects.

## Abbreviations

SCA23: Spinocerebellar ataxia type 23
PDYN: Prodynorphin
ADCA: Autosomal dominant cerebellar ataxia
MSA-P: Multiple system atrophy with predominant parkinsonism
SARA: Scale for the assessment and rating of ataxia
SCD: Spinocerebellar degeneration
ExAC: Exome aggregation consortium
ACMG: American college of medical genetics
